# Feasibility of Mobile Laser Scanning towards Operational Accurate Road Rut Depth Measurements

**DOI:** 10.3390/s21041180

**Published:** 2021-02-08

**Authors:** Aimad El Issaoui, Ziyi Feng, Matti Lehtomäki, Eric Hyyppä, Hannu Hyyppä, Harri Kaartinen, Antero Kukko, Juha Hyyppä

**Affiliations:** 1 Department of Remote Sensing and Photogrammetry, Finnish Geospatial Research Institute FGI, The National Land Survey of Finland, Geodeetinrinne 2, 02430 Masala, Finland; ziyi.feng@nls.fi (Z.F.); matti.lehtomaki@nls.fi (M.L.); eric.hyyppa@gmail.com (E.H.); harri.kaartinen@nls.fi (H.K.); antero.kukko@nls.fi (A.K.); Juha.Hyyppa@nls.fi (J.H.); 2 Department of Built Environment, Aalto University, 02150 Espoo, Finland; hannu.hyyppa@aalto.fi; 3 Department of Geography and Geology, University of Turku, 20500 Turku, Finland

**Keywords:** road rut depth, road mapping, road maintenance, laser scanning, point cloud, MLS, TLS, photogrammetry

## Abstract

This paper studied the applicability of the Roamer-R4DW mobile laser scanning (MLS) system for road rut depth measurement. The MLS system was developed by the Finnish Geospatial Research Institute (FGI), and consists of two mobile laser scanners and a Global Navigation Satellite System (GNSS)-inertial measurement unit (IMU) positioning system. In the study, a fully automatic algorithm was developed to calculate and analyze the rut depths, and verified in 64 reference pavement plots (1.0 m × 3.5 m). We showed that terrestrial laser scanning (TLS) data is an adequate reference for MLS-based rutting studies. The MLS-derived rut depths based on 64 plots resulted in 1.4 mm random error, which can be considered adequate precision for operational rutting depth measurements. Such data, also covering the area outside the pavement, would be ideal for multiple road environment applications since the same data can also be used in applications, from high-definition maps to autonomous car navigation and digitalization of street environments over time and in space.

## 1. Introduction

Road network maintenance takes an extensive share of public expenditure in many countries. In the US alone, the estimated countrywide road maintenance backlog is $420 billion [1]; the corresponding value in Finland is €2.5 billion [2]. The common causes of road distress include overloading, frost, use of studded tires, driving speeds, the thickness of surfacing, traffic volume, the type of surfacing material, improper or poor road surface drainage, lack of proper road maintenance, and improper design. Road distresses disturb traffic flow and safety, and cause an increase in fuel and service costs, time delays with increased pollution, and other trouble for road users.

Early identification of road distress is essential, because it provides invaluable information about the development of damage due to weathering and wear, and also offers an early warning for detecting deeper structural problems due to heavy traffic in combination with climate effects such as increased winter rain, accelerated freeze-thaw cycles and softening road foundation due to excessive water runoff. Thus, improved methodologies to allow faster and objective/reliable pavement condition data for informed and optimized pavement maintenance have potentially enormous economic and environmental benefits. Proper, timely, and selective road maintenance extends the lifespan of the pavement, reduces the cost of road maintenance, lessens vehicle damage and accidents, and minimizes sustained traffic disturbance. An automated, accurate and robust distress detection system is essential to quantify the quality of road surfaces and assist in optimizing the maintenance of the road network. The automatic detection system should be able to detect early pavement degradations, such as transverse and longitudinal cracks, potholes, rutting, road fretting, road deformation, and standing water, for minimized maintenance and timely and informed renovations.

State-of-the-art automated pavement distress detection methods are discussed in multiple reviews, such as [3,4,5,6]. In the past two decades, many research groups have been developing pavement distress detection and recognition algorithms, using 2D passive imaging systems (e.g., [5,7]). Over time, increasing attention has been drawn to the development of active laser-based 3D data acquisition systems for road distress mapping [8,9,10,11,12]. Despite this fact, there are only a few published papers on measuring road rutting using mobile laser scanner (MLS). For example, MLS has been studied for off-road driving capabilities in robotics [13] as an assisting means of navigation. Zhang et al. [14] performed experimental tests for road distress, and briefly mentioned the possibility of rut analysis. Gézero and Antunes [15] applied ten manually-measured road cross-sections, showing that it is possible to measure road rut depths with better accuracy than the nominal 5 mm precision of the MLS system used.

**Figure 1:**
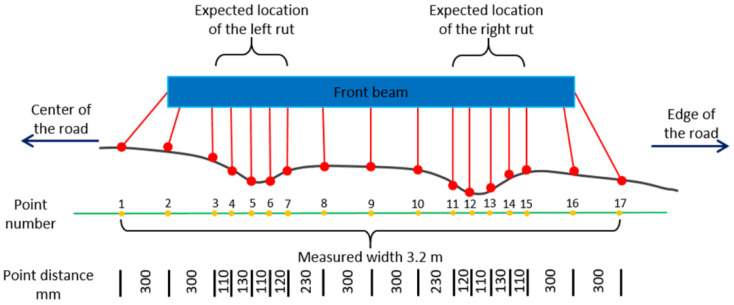
Measurement principle of laser profilometer for rutting measurements. The measurement area is determined by the Finnish standard in the way that laser beams 3–7 should locate at the left rut. Such a system can cover a 3.2-m-wide area of the pavement, with point spacing of 11–30 cm across the road [20].

In the current operational road inspection, human raters travel all over the road measuring its distress elements, but these surveys are too laborious, slow, costly, and unsafe to perform at the scale of the whole network even if prioritized based on road class or traffic load, and they are prone to subjective errors. For example, manual rut depth measurement is performed by placing a straight edge across a rut and the distance between the straight edge and the rut bottom is measured [16] as applied also in Gézero and Antunes [15]. Quantitative analysis of road rut depths is for the most part missing from the scientific literature due to lack of accurate and extensive field reference. Therefore, in the previous rut depth studies, reference has been very limited. Since the phenomena under our scrutiny is affected by road surface roughness (grain size of the gravel used and wear of bitumen) and laser ranging accuracy, statistical in-depth analysis are missing. This has led to the situation that road administrations are mainly using old-fashioned profilometers for rutting measurement in their operations. Profilometer laser systems typically include a fairly limited number of laser detectors (e.g., 13 or 17) installed on a bar perpendicular to the direction of the road [17,18,19], as shown in Figure 1, which further illustrates the operating principle of such a laser profilometer for rutting measurements. The shortcoming of such a special rut measuring system includes a low capability in finding the actual maximum left and right ruts due to limited point spacing across the lane. As a result, despite high range accuracy, such systems tend to underestimate the rut depths [15]. Another shortcoming is that such systems are incapable of measuring any road distress other than ruts. While sparse sampling does not permit detection of other surface defects, it biases individual measurements, in effect leading to false rut depth estimation. Even today, such profilometer systems are considered as de facto reference for new sensors, shown recently by Virtala et al. [19].

Additionally, there are no international standards for rut depth definition and calculation [21]. In many countries, studded tires cause rutting, and yearly rutting measurements are needed to optimize road maintenance frequencies. In Finland, pavements are updated when rutting depth is over 15 mm, when safety of the road is suffering or when correction becomes topical. Main roads with high traffic flows are paved every 2–5 years, whereas suburban and residential streets can have 30–40 year updating cycles. All roads are classified into quality classes based on rut depth, traffic speed and volume. These classes are determined with 1 mm rut depth intervals. Rut depths are provided with 100 or 1000 m averages, and in future with 10 m averages. It can be seen that rut depths should be measured with 1 mm precision at 10 m averages, providing submillimeter accuracy on 100 m averages [22,23].

In our study, we incorporate several innovative elements. We will show that carefully conducted terrestrial laser scanning (TLS) measurements can be used to serve as reference data for rutting studies. To verify the accuracy of the TLS data, three validation areas with approximate sizes of 2.4 m^2^ (a 1-m wide rectangle across the lane) were measured by a stereo-photogrammetric technique, which offers higher accuracy. Besides, a total of 64 one-meter-wide pavement plots across the lane are utilized for the rut depth study, which are large enough to investigate the feasibility of MLS data for rut depth. Operationally, road rut depth measurement results at 10 m intervals will be requested in the near future [2] and we will show that MLS can provide the precision needed in operational nationwide rut measurements. We based our measurements on relatively standard but high-end MLS technology since it provides multiple use of road environment surveying data in applications beyond rutting measurements including navigation, traffic noise modeling and mitigation, nearby city 3D modeling, and in the production of high-definition (HD) maps for the needs of autonomous cars, to be discussed in Section 4.3 of this paper.

## 2. Materials and Methods

### 2.1. Test Site

The survey area was located (60°09′46″ N, 24°32′21″ E) in the municipality of Kirkkonummi, Southern Finland, where a 3-km-long section of a two-lane regional road was selected as a test road. The speed limit in the area is between 40 and 50 km/h. This section of the road was selected because of the condition of the pavement, with a varied state of rut offering a good living laboratory for comparative measurement system analysis and computational methodology. The road pavement at the site exhibits different properties such as new and old asphalt, different depths for ruts, and various types and sizes of cracks and potholes.

### 2.2. Experiment Description

**Figure 2:**
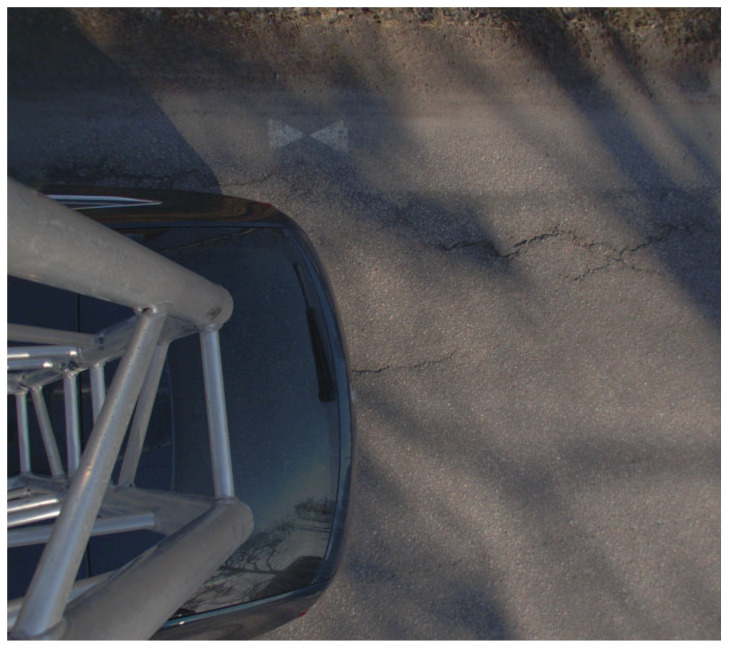
Ground target painted on the pavement. The location for the target was measured to the center between the two triangles.

The road section was signaled by painting white hourglass shaped signal patterns on the road surface at the edge of the carriageway (Figure 2) about every 50 m, on opposing sides of the road. Sixty-two signals were painted per lane, totaling 124 signals altogether. Real-time kinematic (RTK) Global Navigation Satellite System (GNSS) measurements were performed for each signal center to serve as reference points for georeferencing and validation of MLS data, using the Topcon HIPER HR RTK GNSS system (Topcon Positioning Systems Inc., Livermore, CA, USA). The receiver was set to take five measurements per signal to compute the signal position. The purpose of the signals was to allow alignment between the point cloud datasets collected with the different instruments, enabling a comparative analysis.

Because this study focused on evaluating the accuracy of the MLS system in road pavement rut depth derivation, the MLS data had to be compared with other, assumedly more accurate, measurement methods. The purpose of the TLS measurements was to serve as reference data for MLS measurements; the purpose of the photogrammetric measurements was to verify the accuracy of the TLS data.

Since TLS and photogrammetric measurements were static, the evaluation of the TLS instrument was limited to selected study plot areas. The painted signals served as indicators for the test plots, which were defined as one-meter long and 3.5-m (width of one driving lane) wide rectangular sections around the signals for each lane. As a result, in total 64 TLS plots (1.0 m × 3.5 m) were scanned and used in this study for the analysis. Measurements by photogrammetric methods were performed to validate the TLS data on three of the pavement plots covering an approximate area of 2.4 m^2^ each.

### 2.3. Data Acquisition

#### 2.3.1. MLS Data Acquisition

**Figure 3:**
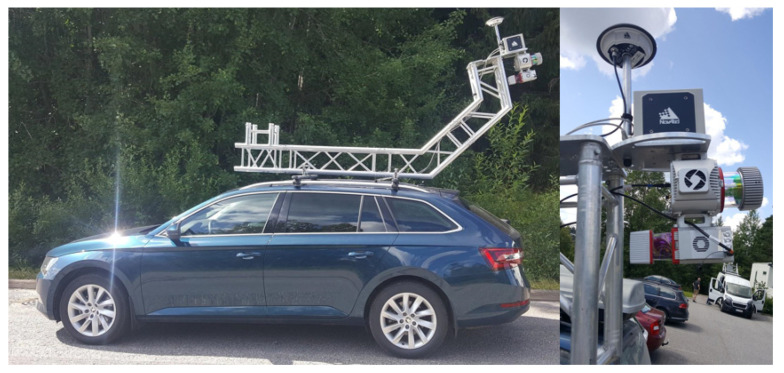
Roamer-R4DW system is a vehicle-mounted mobile laser scanning (MLS) system for road environment mapping. The unit offers unique dual-wavelength sampling of objects. In the image on the right, the Global Navigation Satellite System (GNSS) antenna is on top, with the inertial measurement unit (IMU) and VUX-1HA and miniVUX-1UAV scanners back-to-back to provide cross-track scanning over the road surfaces. The system could also be fitted with a panoramic camera for georeferenced images.

The MLS data were collected in June 2018 using the FGI Roamer-R4DW mobile mapping system developed at the Finnish Geospatial Research Institute (Figure 3). The system provides kinematic 3D laser scanning measurements, and panoramic imagery, at high resolution, enabling mapping and modeling for road asset management. Roamer-R4DW consists of two profiling scanners (the first is used in the experiment in this paper), a positioning system mounted on a modular aluminum truss structure for versatility and a high sensor elevation for enhanced visibility behind street-side objects. The two 2D scanners mounted on Roamer-R4DW were Riegl VUX-1HA and Riegl miniVUX-1UAV (RIEGL Laser Measurement Systems GmbH, Horn, Austria) (referred to as VUX and miniVUX in the following), operating at two distinctive wavelengths, 1550 and 905 nm, respectively. Correspondingly, the beam divergences were 0.5 mrad and 0.5 × 1.6 mrad, while the ranging accuracy specifications were 5 mm and 15 mm for 3 mm and 10 mm precision, respectively.

The positioning subsystem consists of a NovAtel ISA-100C inertial measurement unit (IMU) and a Pwrpak7 global navigation satellite system (GNSS) receiver to obtain the position and orientation of the sensors as a function of time. The IMU output data rate was 200 Hz, and GNSS range observations were recorded at 5 Hz. The trajectory solution is typically computed in multi-pass differential post-processing to obtain the best accuracy possible at the given GNSS constellation during the survey and the environment, specifically in terms of GNSS visibility.

The test area was driven once in both directions for MLS data calibration, but only the data collected on the eastbound lane was used in this study. The average driving speed during the measurements was 40 km/h. The scan settings used were 250 and 100 LPS (lines per second), and 1017 and 100 kHz PRF (pulse repetition frequency) for the VUX and miniVUX, respectively. At the given speed, this resulted in about 44 mm and 111 mm profile spacings for the respective scanner data (i.e., ~22 and ~9 lines per each 1 m wide validation plot). Point spacing along the profile was 4.3 mm immediately below the VUX scanner at a minimum distance of about 2.9 m from the road surface, which corresponded to the actual laser beam spot size at the same distance. The general point density on the road surface was approximately 5000 points/m^2^ in the middle of the lane immediately behind the measurement vehicle. For miniVUX at 2.7 m above the road surface, the corresponding point spacing was 17 mm, and the density was about 500 points/m^2^. Beam size was not specified for short ranges, but was expected to be similar to that of VUX, although slightly elliptical in shape. However, miniVUX data was not used in this study.

#### 2.3.2. TLS and Photogrammetric Measurements

**Figure 4:**
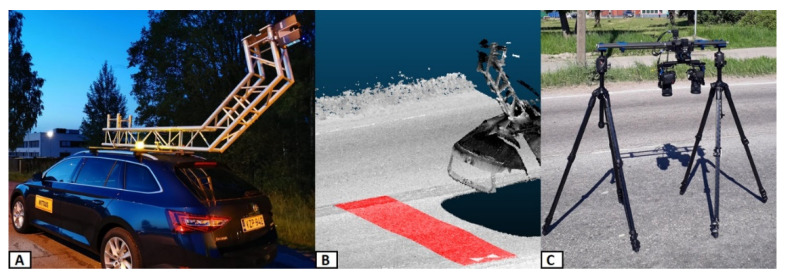
(**A**) Measurement system for TLS. (**B**) TLS point cloud from one measurement. (**C**) Photogrammetric measurement system.

TLS measurements were performed using the FARO Focus S 350 (FARO Technologies, Inc., Lake Mary, FL, USA). The scanner uses the phase-shift principle for measuring distances. Its measurement speed is up to 976,000 points/second. It has a ranging accuracy of ± 1 mm, and it uses 1550 nm wavelength. The field of view (FOV) for the scanner is 360° in the horizontal axis, and 300° in the vertical axis. For this study, scanning parameters were set to provide a point spacing of 7.7 mm in both vertical and horizontal directions at a distance of 10 m from the scanner, resulting in 2.3 mm point spacing on the plot road surface. The measurement time for a single scan was approximately three minutes. The scanner was mounted on the roof rack of the car so that the blind spot of the scanner was pointing in the driving direction, allowing full visibility of the road surface below the scanner (see Figure 4A). During the TLS measurement, the vehicle was stationary in the center of the lane, placing the scanner about 2.8 m above the plot (Figure 4B). The aim of this setup was to get the scanner as close as possible to the plot area so that the point density remained as high as possible and to ensure that the whole plot was fully visible for the scanning. The measurement was then repeated for each plot. The point density for the measured plots was about 150,000 points/m^2^. Stationary TLS measurements were performed at night between 11 PM and 5 AM, when the traffic flow was minimal, and there was no heavy vehicle traffic. This was to ensure that passing vehicles did not cause the measuring system to move, and measurements could be carried out with minimum disturbance to the public.

Photogrammetric measurement was conducted with a custom-built and automatically operated stereo camera system (Figure 4C). The system was built around Nikon D850 cameras equipped with Sigma Art 50 mm 1.4 G prime lenses. The cameras were mounted on a remotely controlled and electronically operated camera rig. The average ground sampling distance (GSD) of the three plots varied between 0.079 and 0.100 mm. The mean reprojection error varied between 0.090 and 0.116 pixels. All the image blocks were processed without 3D control points. The scale of the blocks was determined by utilizing eight scale bars in each block. The sigma of the scale varied between 0.014 mm and 0.021 mm. 3D point densities on the test plots varied between 163 and 230 points/mm^2^.

### 2.4. MLS Data Pre-Processing

The pre-processing of the MLS data had three steps: (1) trajectory computation; (2) MLS system calibration; and (3) point cloud georeferencing.

The trajectory, i.e., the measurement path of the MLS, was computed using the NovAtel Waypoint Inertial Explorer (version 8.80) (NovAtel Inc, Calgary, AB, Canada). For the differential GNSS, we used a virtual GNSS base station generated at the site from the Trimnet service (Geotrim Oy, Vantaa, Finland). The 1 Hz virtual base station data were interpolated to meet the 5 Hz records of the Roamer-R4DW positioning system. A 15-degree elevation angle was used to reduce multipath effects in the GNSS signals, and the static initialization method was used for the IMU. The final trajectory was computed in a multi-pass process with three forward and reverse computations, and the solutions were combined and smoothed for the final result.

The trajectory data were associated with the raw laser scanning data (18 leap seconds were also applied to match the time systems), and initial system internal bore-sight parameters (three translations and rotations between the system IMU and each scanner) were applied. The initial point cloud was then generated using RiProcess software. After this step, we ran a scan alignment tool in the RiProcess to improve the initial bore-sight rotations for mounting, but the translations were considered as known parameters. Robust distance weighted estimation was used to solve the parameter values for planar features automatically extracted from the point cloud data and matched at locations with data from multiple visits. After solving the fine-tuned orientation bore-sight parameters, we generated the final point cloud for rut study method development and rut depth analysis.

The trajectory accuracy was estimated in the post-processing software by computing the mean, standard deviation, and the root mean square (RMS) in the 3D position and the attitude (square root of the mean value of the squared observed values, e.g., differences in position and attitude between the forward and reverse trajectory solutions to their combined and smoothed solution) over the timespan of the data. The estimated position mean accuracy was 4.5 mm with 1.2 mm standard deviation. The corresponding RMS was 4.6 mm. The average attitude accuracies were −0.0711, −0.0687, and −0.3139 arcminutes for Roll, Pitch, and Yaw, respectively. The corresponding standard deviations were 0.0584, 0.0286, and 0.2193 arcminutes, and the RMS values 0.0920, 0.0744, and 0.3829 arcminutes for Roll, Pitch, and Yaw. The Yaw/Heading angle uncertainty dominates the solution, but the effect of this at a range of 10 m from the scanner (Yaw RMS) results in 1.1 mm point displacement, and the maximum absolute uncertainty (0.8179) yields a 2.4 mm spatial error.

**Figure 5:**
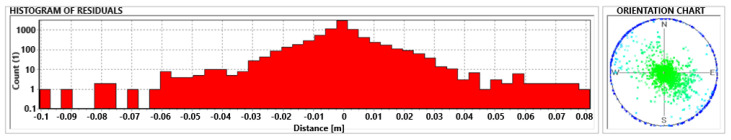
Histogram of residuals and orientation chart of the bore-sight calibration result show good accuracy and reliability of the solution.

The bore-sight calibration resulted in 7800 planar pair matches and, eventually, a 10.2 mm error (standard deviation) in the estimation. The values applied to the final data georeferencing were −0.03501, −0.03877, and −0.18758 for Roll, Pitch, and Yaw, respectively. The plot of the histogram of the residuals (Figure 5) shows that the majority of the observations were within 30 mm around the mean (note the logarithmic scale of the plot), and the orientation of the observations was well distributed for a reliable solution.

The pre-processed MLS data were compared against RTK measurements at the targets on the road, clearly visible in the reflectance data from the scanners, and the study concluded that the MLS positioning system was sufficiently accurate for georeferencing. The RTK measurements were used to confirm the success of the georeferencing with the MLS system. Subsequently, the data measured by the other instruments for the study were georeferenced with the MLS data: the TLS data were georeferenced in CloudCompare (version 2.10.2, http://www.cloudcompare.org/ (accessed on 15 January 2021)), using the georeferenced MLS data, and the photogrammetric data were aligned with the georeferenced TLS data using the same software.

**Figure 6:**
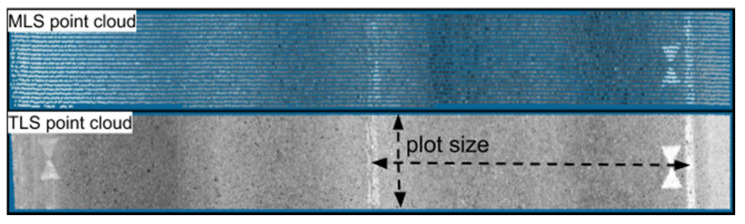
MLS (**above**) and TLS (**below**) point clouds from the plot area (Plot 2). Here, the scanner was located on the right lane in both cases. The right lane was therefore the studied plot area.

Test plots were created in Bentley MicroStation (v.10.00.00.25) by cutting the data from the road surface into one-meter wide slices (Figure 6) according to the MLS scan profiles. The scan profile pattern of the pavement seen at the top of Figure 6 was formed when the scanner mirror rotated at high speed (each mirror rotation creates one scan profile) while the car was moving forward. The scan plane was slightly tilted to a 15-degree nominal angle, as can be seen in Figure 3. The rotation speed of the scanner and driving speed determined the space between two scanning profiles. The number of scan profiles within the plots therefore varied between 19 and 22. An illustration of the scan profiles from Plot 2 can be seen in Figure 6, where the MLS point cloud contains 22 scan profiles for the particular plot.

### 2.5. TLS Preprocessing and TLS Accuracy as Reference

To evaluate the accuracy of the TLS measurement, the photogrammetric and TLS data were aligned using CloudCompare’s registration tools, followed by the use of the Cloud-2-Cloud (C2C) distance comparison tool. C2C is a method in which distances between predetermined reference point clouds and corresponding point clouds are calculated. The method can be used to detect changes between point clouds, and it can also be used to evaluate accuracy when the method is combined with CloudCompare’s Local Statistical Test (LST) tool. Here, the C2C tool calculates distances between two point clouds from the same area, after which the LST tool draws a histogram of the distances between each point pair and calculates a Gaussian distribution, providing the mean distance and standard deviation for each plot.

The RMS value for TLS and MLS data alignment (3D distances between the two datasets) for each plot was between 4 and 7 mm, depending on the road environment; georeferencing was usually more accurate when there were also corresponding points from objects above the pavement, such as streetlamps, fences, and road signs. TLS and photogrammetric datasets were aligned using the same method as TLS and MLS. Here, the RMS value for registration was around 0.5 mm for all the three validation plots.

**Figure 7:**
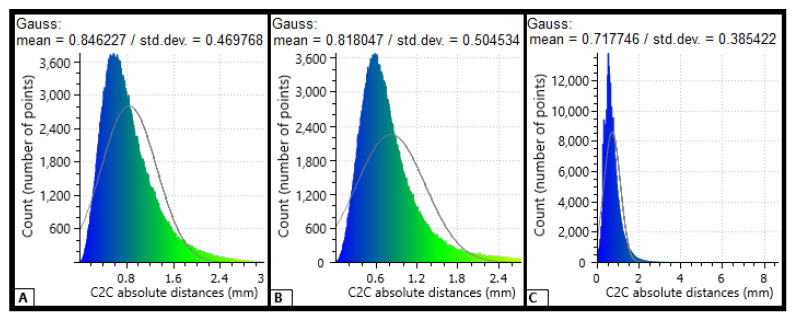
Distances between TLS and photogrammetric point cloud points. The *y*-axis represents the number of corresponding points, and the *x*-axis represents the absolute distance (in millimeters) between corresponding points. (**A**) (Plot 1): Cloud-to-cloud absolute distance for 390,000 points (road surface of 2.4 m^2^). (**B**) (Plot 2): C2C absolute distance for 390,000 points (road surface of 2.4 m^2^). (**C**) (Plot 3): C2C absolute distance for 330,000 points (road surface of 2.2 m^2^).

The TLS accuracy was evaluated using photogrammetric data and by using the cloud-to-cloud (C2C) distance tool in CloudCompare. The mean absolute C2C distances between the TLS and photogrammetric point cloud were 0.85 mm (±0.47 mm, standard deviation) for Plot 1, 0.82 mm (±0.50 mm, standard deviation) for Plot 2, and 0.72 mm (±0.39 mm, standard deviation) for Plot 3 (Figure 7). Thus, better than 0.5 mm precision for TLS was confirmed.

**Figure 8:**
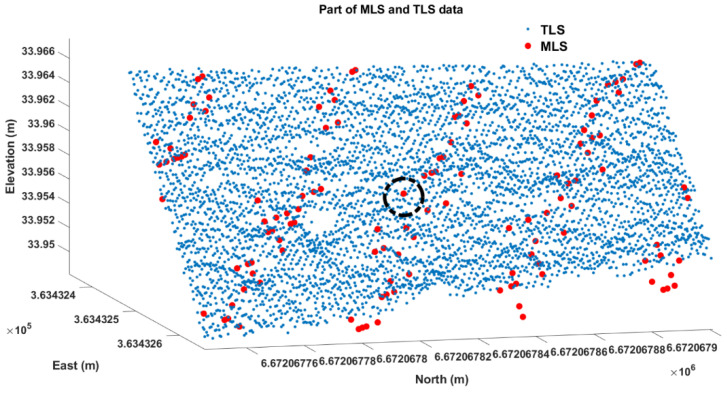
A piece of road surface from the MLS (red dots) and TLS (blue dots) data.

The TLS scan pattern was much denser than the MLS point cloud (see Figure 6 and Figure 8 for reference) and, as the 3D scan was stationary, the scan pattern on the ground in general differed from that of the MLS. The normally vertical rotation axis of the TLS scanner was tilted 75 degrees backward, and while the scanner rotated across the lane, the scanning mirror cast scan profiles roughly oriented along the longitudinal axis of the road. Given the scan settings and the approximate 2.8 m distance to the road surface, the average point spacing within the plot was approximately 2.3 mm.

**Figure 9:**
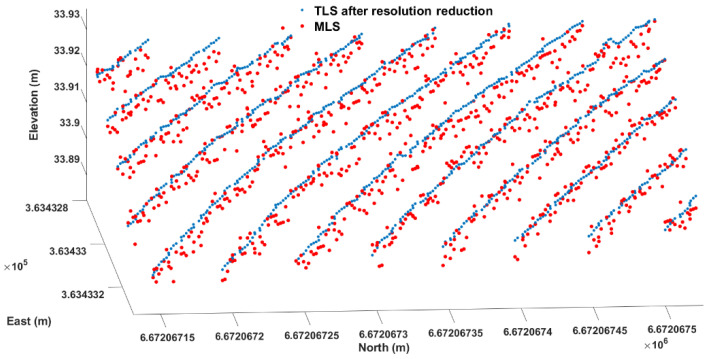
A piece of road surface from MLS (red dots) and down sampled TLS (blue dots) data.

To generate a high-accuracy rut depth reference, down-sampling of the TLS data was performed to generate road profiles at the locations of the profiles generated by the MLS. The down-sampling was conducted by averaging the neighboring TLS data around the MLS data. More specifically, at the position of each MLS point, the surrounding TLS points were selected within a horizontal distance of 5 mm (black circle in Figure 8). The z coordinates of the down-sampled TLS points were the average elevation values within the neighborhood, and the horizontal coordinates were equal to those of the corresponding MLS data. In this way, the rut depth estimation with TLS data and MLS data can be performed at exactly same locations, which help avoid possible variation errors introduced by location. The down-sampling matches TLS data with MLS data to guarantee accurate TLS reference (Figure 9).

### 2.6. Applied Signal Processing and Statistical Methods

A digital low-pass filter, which only allows a signal with a frequency of less than the preset cut-off frequency to pass [24,25], was applied to reduce data noise, therefore eliminating short-term fluctuation in the ruts. Low-pass filters can be designed with various algorithms, and in our study a finite impulse response filter with a Hamming window was applied [26], in which the two most important factors were the filter order and the cut-off frequency. Similar filters were applied to both the MLS and the down-sampled TLS data, while the filter order, which determined the filter window length, was 25 for the MLS data, and 15 for the TLS data.

In addition, some statistical tools were used to assess the performance of the MLS rut depth analysis. The bias, random error, and root-mean-square error (RMSE) of a variable x are evaluated using the following equations:

(1) $$\mathrm{bias}=\frac{1}{N}\sum_{i=1}^{N}\left({\hat{x}}_{i}-x_{i}\right),$$

(2) $$\mathrm{random}\;\mathrm{error}=\sqrt{\frac{1}{N-1}\sum_{i=1}^{N}{\left(e_{i}-\mathrm{bias}\right)}^{2}},$$

(3) $$\mathrm{RMSE}=\sqrt{\frac{1}{N}\sum_{i=1}^{N}{\left({\hat{x}}_{i}-x_{i}\right)}^{2}},$$

where N is the number of observations, ${\hat{x}}_{i}$ is the observation, and $x_{i}$ is the reference value. Variable $e_{i}$ is the difference between the observation and reference value.

The corresponding relative bias (${\mathrm{bias}}_{\mathrm{rel}}$) and RMSE (${\mathrm{RMSE}}_{\mathrm{rel}}$) are defined as follows:

(4) $${\mathrm{bias}}_{\mathrm{rel}}=\frac{\mathrm{bias}}{\bar{x}}*100\%,$$

(5) $${\mathrm{RMSE}}_{\mathrm{rel}}=\frac{\mathrm{RMSE}}{\bar{x}}*100\%,$$

where $\bar{x}$ is the mean reference value, which is defined as

(6) $$\bar{x}=\frac{1}{N}\sum_{i=1}^{N}x_{i},$$

## 3. Computational Algorithms

**Figure 10:**
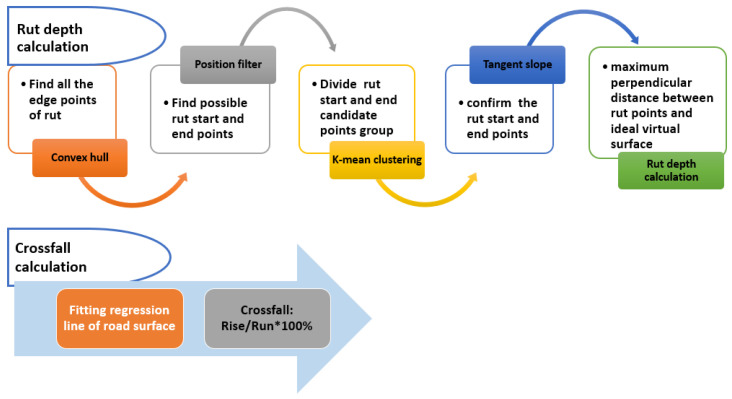
The process of rut depth and crossfall calculation.

In this section, the computational methods for the rut depth and crossfall calculation are described. Figure 10 briefly presents the process for obtaining the rut depth and the crossfall, while the details of each process block are explained in the following sections.

### 3.1. Rut Depth Estimation

**Figure 11:**
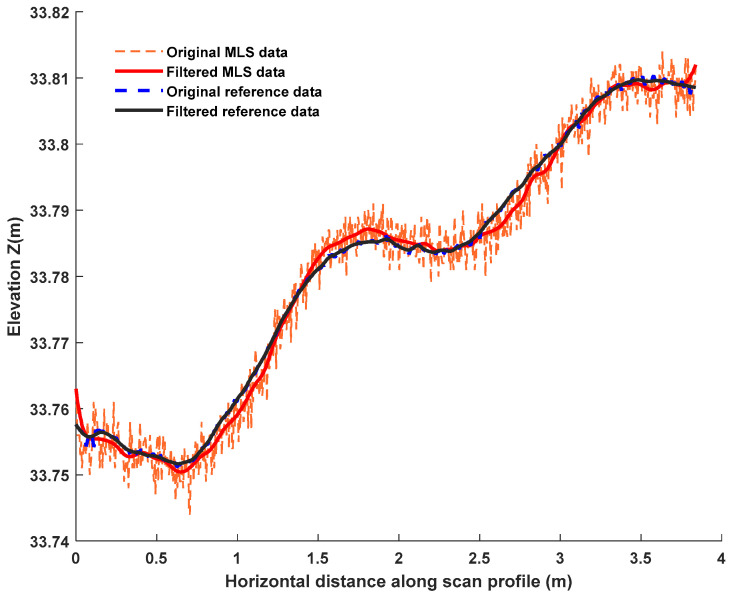
Comparison of MLS and reference data for one scan profile.

To guarantee the accuracy of the rut depth and crossfall estimation, the points of each profile were transformed into a new local scan-profile-specific 2D coordinate system. In more detail, the horizontal axis corresponded to the horizontal distance along the scan profile with respect to the first point on the scan profile. The vertical axis coincided with the original z axis. Figure 11 illustrates one filtered (see Section 2.6) scan profile of the MLS and reference data in the new local scan-profile-specific coordinate system.

In the Nordic countries, rut depths are estimated using the wire method [20,27]. The wire method determines the rut depth as the maximum distance between a tensed wire and a road surface measurement point on a profile (see, e.g., Figure 1).

**Figure 12:**
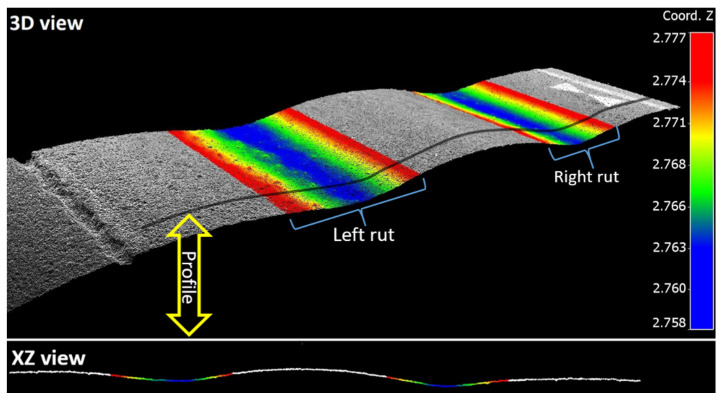
TLS point cloud from test plot that illustrates left and right ruts (rainbow colors). Below is a side view that shows one TLS profile extracted from the plot.

For each plot, rut depths were estimated from each scan profile independently, and the rut depths were then averaged over all the scan profiles in the plot, resulting in the final rut depths of the plot. Each scan profile was a cross section of the whole lane, thus containing two ruts in the left and right parts respectively (Figure 12). The depths of the left and right ruts, as well as the maximum rut, were determined in accordance with the standards of the Nordic countries [20,27].

**Figure 13:**
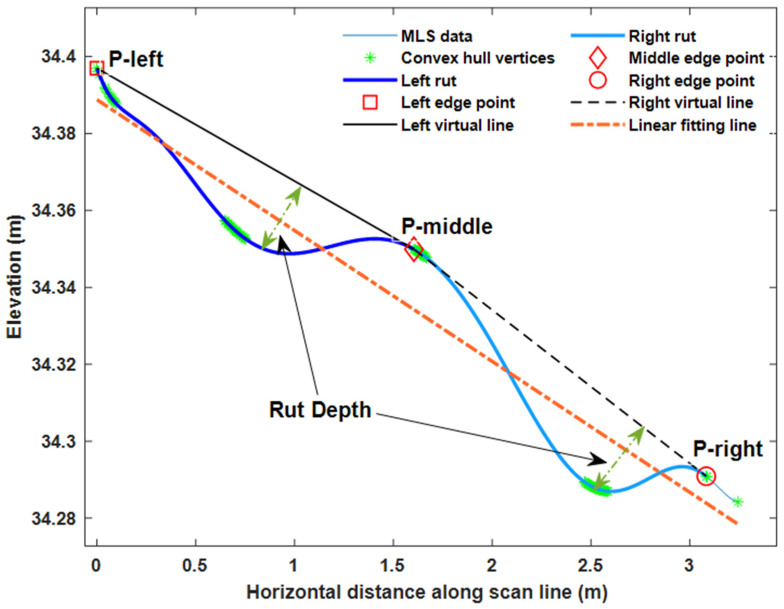
Left and right rut depth calculation for one scan profile in MLS data.

Figure 13 presents how the rut depths were estimated in one scan profile. For each rut, an ideal road virtual line without rutting was represented by a line passing through start and end points of the rut (black solid line and dash line in Figure 13). The selection of the start and end points of the rut is described below. All points between the rut start and end points were selected, and the perpendicular distances from each selected point to the virtual line were calculated. The maximum distance was used as a rut depth estimate.

To find the start and end points of the ruts, a convex hull of the road data for each profile was computed. The vertices making up the convex hull (green stars in Figure 13) were first classified into upper and lower hull points, and then the upper hull points were further assigned into left, middle, and right groups, according to their locations. More specifically, a least squares fitting line for the whole profile was generated (orange dash dotted line in Figure 13), and all the vertices located above the fitting line were selected. The selected vertices were then assigned into the left, middle, and right groups based on cluster center position and using k-means clustering. The vertex groups acted as candidates for the start and end points of the ruts.

It was assumed that the start and end points of the ruts corresponded to the local minima of the absolute value of the tangent slope along the profile. We sought three local minima, one from each of the left, middle, and right groups (red square, diamond, and circle in Figure 13), which are referred to as the left, middle, and right edge points, respectively, in the following. The left edge point was the start point of the left rut and the right edge point was the end point of the right rut. The middle edge point was both the end point of the left rut and start point of the right rut.

The tangent slope for point $\left(x_{i},y_{i}\right)$, where $i\in I_{\mathrm{prof}}=\left\{1,\cdots ,n\right\}$, where $I_{\mathrm{prof}}$ is the index set of all points on the profile and n is the number of points on the profile, was estimated using the following formula:

(7) $$m_{i}=\frac{y_{i}-y_{i-1}}{x_{i}-x_{i-1}}$$

Let $I_{\mathrm{left}}$, $I_{\mathrm{middle}}$, and $I_{\mathrm{right}}$ denote the index sets of the points in the left, middle, and right vertex groups, that is, $I_{\mathrm{left}},I_{\mathrm{middle}},I_{\mathrm{middle}}\subset I_{\mathrm{prof}}$. The indices of the left, middle, and right edge points, denoted by $i_{\mathrm{left}}$, $i_{\mathrm{middle}}$, and $i_{\mathrm{right}}$, respectively, were retrieved using the following equations:

(8) $$i_{\mathrm{left}}=\underset{i}{\mathrm{argmin}}\;{\left\{\left|m_{i}\right|\right\}}_{i\in I_{\mathrm{left}}}$$

(9) $$i_{\mathrm{middle}}=\underset{i}{\mathrm{argmin}}\;{\left\{\left|m_{i}\right|\right\}}_{i\in I_{\mathrm{middle}}}$$

(10) $$i_{\mathrm{right}}=\underset{i}{\mathrm{argmin}}\;{\left\{\left|m_{i}\right|\right\}}_{i\in I_{\mathrm{right}}}$$

In a real situation, the road geometric shape varies in distinct files, and the points around the peaks are sometimes not contained in the data due to data segmentation, which leads to an issue that the slope of the tangent at the edge point is found not to be the smallest. Consequently, before calculating the rut depth, the start and end points of the rut were verified to avoid errors in the selection of edge points. In other words, the other non-selected convex hull points in the left group were checked as to whether they were below the line composed by the start and end points of the left rut. If not, the start point was changed to the neighboring point, and the rest of the points in the instance were checked again with the newly assigned start and end points. The same verification was also performed for the middle and right rut point groups.

**Figure 14:**
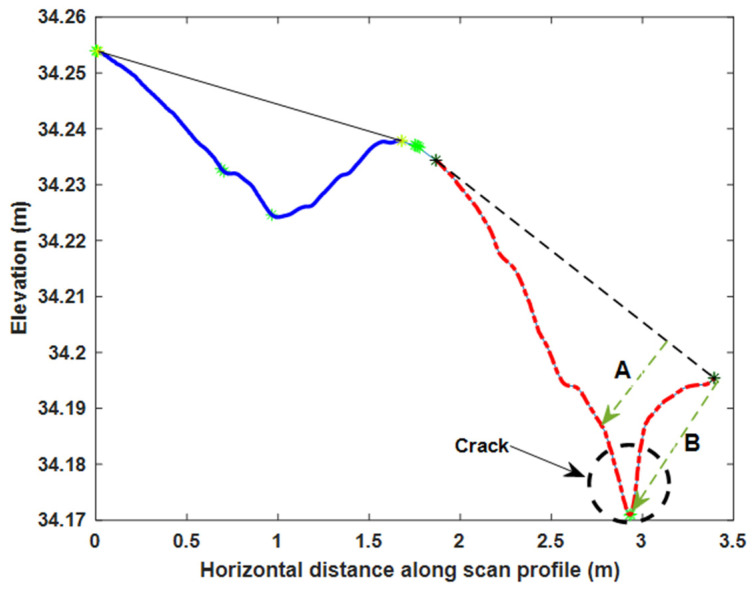
Rut and crack of one scan profile in MLS data.

When conducting the rut depth study, one issue that needed consideration was presence of cracks in the pavement. In some cases, the road surface pavement not only had ruts, but also cracks (or other such damage in general) like the one on the right rut shown in Figure 14. Using the implemented rut depth calculation algorithm, the rut depth for the right rut was observed as B, which was obviously affected by the presence of a crack, while the rut depth determined by A was the actual rut depth. As this paper focuses on rutting, only plots with small amounts of cracks were adopted for the analysis of the performance and robustness of the rut depth estimation method. The crack detection was performed in all the plots automatically using a method developed for the purpose, though we will talk more about crack detection algorithms in a subsequent paper.

Besides the plots spoiled with cracks, there were also plots where the pavement had no obvious ruts, i.e., smaller in depth than the detection capability of the MLS data. As this paper focuses on the rut depth measurement accuracy, these good plots were excluded after they had been identified. Consequently, a total of 34 out of 64 plots was adopted for the rut analysis in this study. As the left and right ruts were treated separately, and the rut depths were averaged over the profiles of each plot, we had a total of 68 rut depth samples from the 34 plots.

### 3.2. Crossfall

Crossfall is an important geometric road surface safety indicator. It is defined as a transverse slope along a horizontal distance [20]. In normal situations, the elevation of the road surface is planned to be highest at the middle, and the slope drains towards the edges. The road slope should be neither too small nor too large; the former slows the rainwater run-off from the road surface, while the latter may result in high heavy vehicle roll vibration. In Finland, the crossfall design is ±15%.

**Figure 15:**
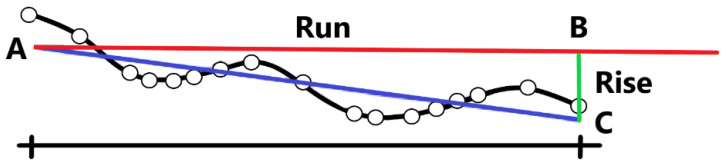
Crossfall measurement method.

Different measurement methods are used for crossfall [28]. In Finland, this is calculated based on linear regression [20]. For a group of 17 measurement points, inherently from the pavement survey system, a regression line is fitted, based on the least square method (line AC in Figure 15). The line is then compared with the horizontal line, and the lateral slope is calculated. In detail, for any point (C) on the regression line, the perpendicular distance from the point to the horizontal line is called Rise (|BC|), and the distance from the start point (A) of the fitting line (AC) to the cross point (B), where the rise perpendicular interacts with horizontal line is called Run (|AB|). The crossfall is then calculated and expressed as a percentage:

(11) $$\mathrm{crossfall}=\;\frac{\mathrm{rise}}{\mathrm{run}}*100\%,$$

**Figure 16:**
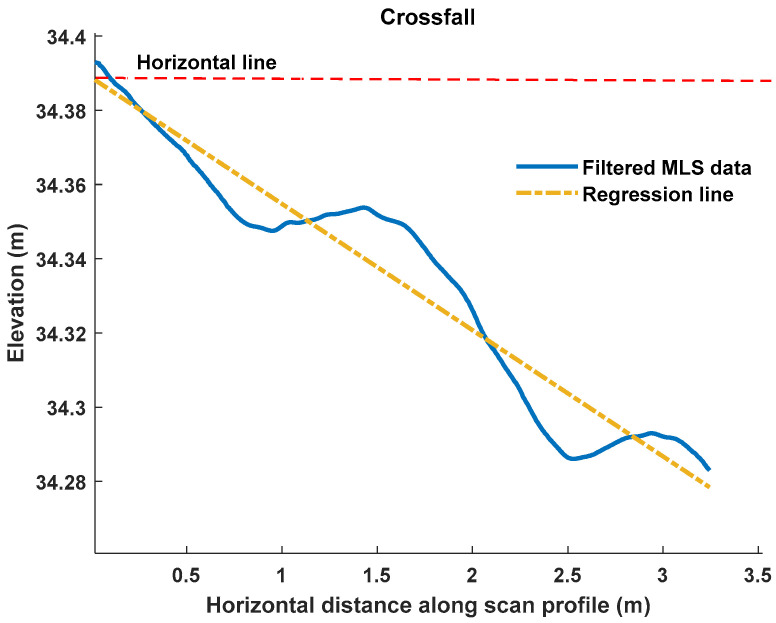
Crossfall estimation for one scan profile in MLS data.

When the crossfall was measured using MLS and TLS data, a regression line was fitted to each profile using the least squares method. One example using MLS data is presented in Figure 16. Then the slope of the fitting line was calculated and converted into percentages according to Equation (11). Similar to the rut depth estimation, crossfall values were averaged over all the profiles for each plot. However, there was only one crossfall measurement, in contrast to the two ruts in each plot. Therefore, 34 crossfall values were acquired in total from 34 plots.

## 4. Results and Discussion

### 4.1. Rut Depth Analysis

**Figure 17:**
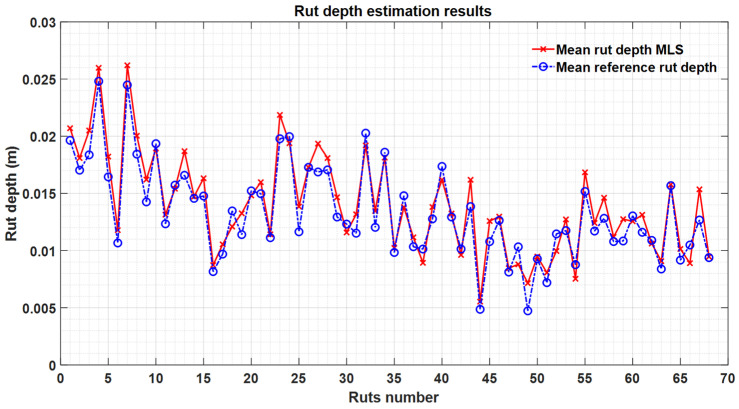
Rut depth estimation results.

Figure 17 presents the accuracy of the rut depth estimates. The rut depths ranged from 4.7 mm to 26.2 mm, and for most rut samples the residuals were small—on average 0.66 mm.

**Figure 18:**
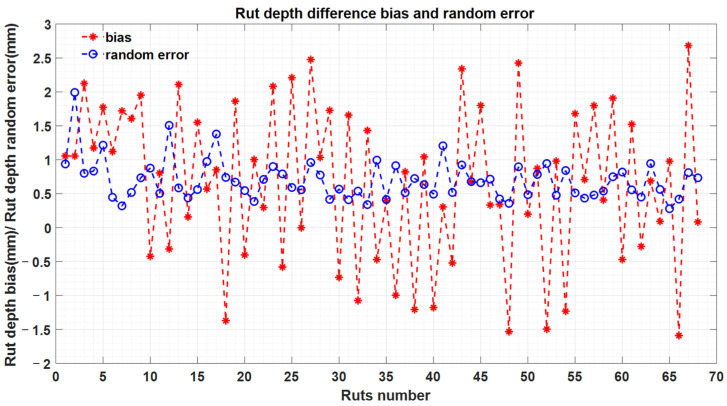
Bias and random error of the rut depth estimation.

Figure 18 demonstrates the bias and random errors inside each rut, calculated from the profiles of the plot. The random errors ranged from 0.28 mm to 2 mm. In addition, the biases from all the 34 plots were between −1.6 mm and 2.7 mm. Bias is mainly caused by the variability of the road surface (grain size, small differences in georeferencing) and how bias can be decreased should be dealt with in future studies. For the 68 sample ruts, the average bias of the rut depth estimation was 0.66 mm, with a 1.4 mm random error, and the RMSE was 1.5 mm. This proves that the rut detection and depth measurement with the MLS data point density, 5 mm ranging accuracy and 3 mm precision specification was sufficiently accurate for the road rutting measurement.

**Figure 19:**
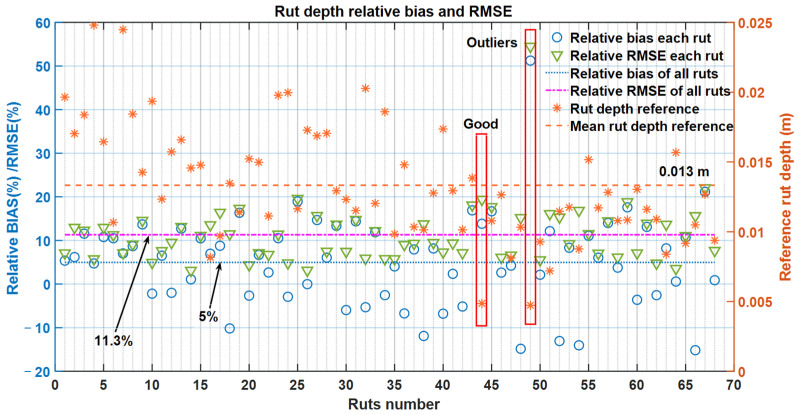
Individual and overall relative bias and root-mean-square error (RMSE) of all the ruts.

Figure 19 presents the relative bias and RMSE for the individual ruts. The ${\mathrm{bias}}_{\mathrm{rel}}$ and the ${\mathrm{RMSE}}_{\mathrm{rel}}$ over all the plots were 5.0% and 11.3%, respectively. The highest ${\mathrm{bias}}_{\mathrm{rel}}$ and ${\mathrm{RMSE}}_{\mathrm{rel}}$ occurred at the same rut (in the red rectangle marked with ‘Outliers’ in Figure 19), which were 51.2% and 54.5%, respectively, and the corresponding reference rut depth was only 4.8 mm. However, at the 44th rut (in the red rectangle marked with ‘Good’ in Figure 19), the reference rut depth was 4.9 mm, but the relative bias and RMSE were 13.8% and 19.4%, respectively, which were much better than the outliers. The relative bias and RMSE are not important when the measured rut depth is less than 10 mm. In Finland, maintenance is targeted when rut depth exceeds 15–20 mm.

### 4.2. Crossfall Analysis

As mentioned above, the elevation of the road surface is usually designed to be the highest at the middle, and the pavement surface drains towards the edges (Figure 16). However, when pavement settlement happens, the elevation of the road surface is changed, leading to crossfall with distinct angles. We therefore assumed that the crossfall was zero when the elevation of the road surface remained the same along the horizontal axis from the middle position of the road to its edge. We also assumed that the crossfall was a positive value when the elevation was lowest in the middle of the road and rose to the edge. Otherwise, the crossfall was a negative value, which fulfilled the design purpose.

**Figure 20:**
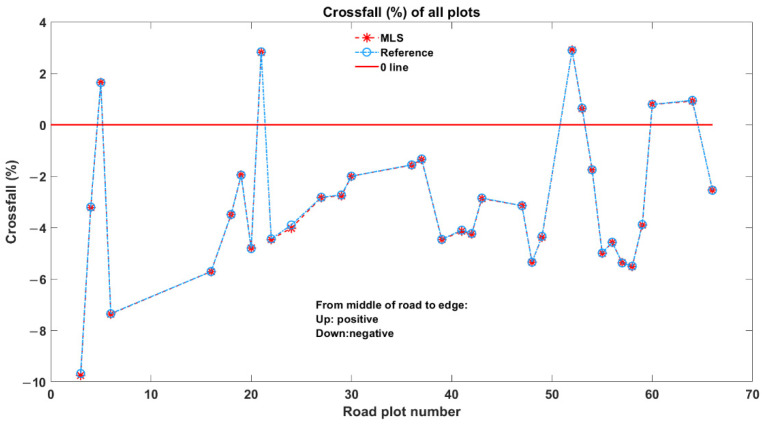
Crossfall estimation results from MLS (red dashed starred line) and reference (blue dashed circle line).

As expected, the calculated crossfalls of all the profiles within each plot rectangle were similar. They were therefore averaged as the final crossfall estimation for each particular plot. The mean crossfall of all plots calculated from both the MLS and reference data are presented in Figure 20. The crossfalls measured from the MLS data matched well with the reference. The maximum and minimum errors were 0.02% and −0.12%, respectively. Moreover, the crossfall bias was −0.0153%, and the random error was 0.0257%.

### 4.3. Discussion

Rutting is the most significant distress caused to asphalt pavement in the Nordic countries because of the use of studded tires in the winter season. Rutting can significantly deteriorate road safety since rainwater may fill ruts, leading to a loss of wheel grip and vehicle traction. Maintenance operations to remove ruts should, therefore, be timely. According to Virtala et al. [2], the rutting rate during winter is 0.5–1.3 mm/year on average, depending on the traffic volume class. Similarly, excessive compacted snow on the road surface could lead to centimeters deep temporary ice ruts with similar effects.

Currently, the most commonly used standard in rutting measurements relies on laser systems with a discrete number of laser detectors installed on a vehicle-mounted bar perpendicular to the direction of the road. From such discrete measurements, the most common rutting features calculated are the maximum rut, the slope, the left and right ruts, the distance between ruts, the ridge, and the cross-sectional area of ruts. In addition, Virtala et al. [2] proposed ratio indicators such as the ratio of the left rut to the right rut, the ratio of the maximum rut to the ridge, and the ratio of the rut and the ridge to the area. Such features could be used, e.g., in change-based rutting studies. Precisely georeferenced MLS data provide good possibilities for future studies on change-based rutting development and evolution of other deterioration of paved surfaces.

There are multiple error sources regarding the measurements. Due to the actual roughness of the road surface, correct elevation level is non-trivial to determine. Therefore, in the current process using a profilometer, there was a bias found in measurements [15], allegedly because not enough samples were taken. Another error source is the laser ranging accuracy. When using an MLS system, the major error source is the laser ranging error, which potentially causes both bias and random errors. Since there are hundreds of samples from the road cross-section, it is possible to calibrate the bias provided that there is an accurate reference to be used. Since rutting measurements are conducted annually, the key parameter is precision (the variation within a repeated observation). Further averaging at 10 m or 100 m intervals reduces the obtained random errors. At best, 100 m statistics reduces the random errors obtained for one-meter-plots shown in the paper by 90%, if error sources are independent.

In our study, the bias was 0.66 mm, while the random error and RMSE were 1.4 mm and 1.5 mm, respectively, showing that sub-millimeter accuracies can be obtained with 100 m averages. On the other hand, using MLS, Gézero and Antunes [15] reported rutting RMSE of 3.2, 5.6, 7.9, and 8.3 mm, with different computational strategies.

There are also high-end pavement measurement systems under development allowing the measurement of thousands of points in each profile with high ranging accuracy (at best, submillimeter level), but they are still very expensive, cover only the road surface and therefore do not allow multipurpose use of the data. Such systems are provided, e.g., by Pavemetrics (Québec, QC, Canada) and Fraunhofer Institute (Munich, Germany).

Currently, there are multiple needs to map roads and the road environment. In Finland alone, measurements for multiple services and geospatial information systems are performed on the same roads separately. The national Digiroad covers the center lines of the roads and streets with key attribute information. Ruts are measured with dedicated service cars, equipped with profilometers. Other distresses are monitored separately by human recognition.

The road environment is increasingly being mapped using MLS for navigation, traffic noise modeling and mitigation, nearby city 3D modeling, and other applications. MLS can even be used to produce high-definition (HD) maps for the needs of operating autonomous cars in the future. In many cases, HD maps consist of the point clouds of the environment, captured with autonomous vehicles or high-end mobile mapping systems. Autonomous positioning is performed using matching algorithms, such as variants of iterative closest point techniques [29,30,31,32].

To decrease the costs of such road and road environment surveys, and increase the multiple use of road environment surveying data, it would be beneficial to be able to conduct all imaginable road maintenance related measurements with a single mobile laser scanning (MLS) survey.

## 5. Conclusions

To decrease the costs of road and road environment surveys, and increase the multiple use of road environment surveying data, it would be beneficial to be able to conduct all road related measurements with a single MLS survey. In this paper, the pavement rut depth and road slope measurement capability of an MLS system was investigated using terrestrial laser scanning (TLS) measurements as a reference. To verify the accuracy of TLS data, the TLS data were analyzed with geometric models obtained with stereophotogrammetry, resulting in standard deviations less than 0.5 mm for the verification plots. The MLS and TLS data were collected in a short time interval, and in total 34 one-meter-long road plots were qualified for evaluation in the end. Using the implemented fully automatic rut analysis, we found that the bias and random errors were 0.66 mm and 1.4 mm for the rut depth estimation using Roamer-R4DW MLS (based on Riegl VUX-1HA). The mean error of the crossfall measurements of the road surface was −0.0153%, with a standard deviation of 0.0257%.

The results proved that rut depth and crossfall slope measurements using MLS are highly accurate and feasible for operational application in many countries especially in busy roads where maintenance cycles are short. This offers the potential to develop a mobile road surface management system supporting various road environment applications with a high level of automation, and the possibility to use commercial off-the-shelf (COTS) LiDAR sensors for road asset inventories.

## Data Availability

Not applicable.
